# Undiagnosed Coronary Artery Disease in Patients with COPD

**DOI:** 10.3390/jcm15051896

**Published:** 2026-03-02

**Authors:** Zsófia Éreth, Márta Papp, Réka Faludi, Erzsébet Juhász, Enikő Horváth, Attila Kónyi

**Affiliations:** 1 Heart Institute, Medical School, University of Pécs, 7624 Pécs, Hungarykonyi.attila@pte.hu (A.K.); 2 Hospital of Mohács, Medical School, University of Pécs, 7700 Mohács, Hungary; 3 Pécs Diagnostic Center, 7623 Pécs, Hungary; 4 Faculty of Medicine, Medical School, University of Pécs, 7624 Pécs, Hungary

**Keywords:** coronary artery disease, chronic obstructive pulmonary disease, inhaled corticosteroid therapy

## Abstract

**Background:** Coronary artery disease (CAD) commonly coexists with chronic obstructive pulmonary disease (COPD), but may be under-recognised, since symptoms such as dyspnoea and chest discomfort are often attributed to lung disease. We hypothesised that coronary artery disease is highly prevalent in patients with COPD, even in the absence of typical angina symptoms. **Methods:** This study aimed to detect CAD in patients with COPD. We conducted a single-centre observational study, including 76 patients with no known previous cardiovascular events. To detect ischaemic heart disease, three methods were used, according to standard clinical indications: coronary angiography, coronary CT, and calcium score analysis on chest CT. The findings were categorised according to lesion severity and vessel involvement. **Results:** A substantial proportion of patients with COPD harboured previously undiagnosed atherosclerotic coronary disease (78%). However, most detected disease was non-obstructive atherosclerosis (56%), whereas severe stenosis was present in approximately one-third of patients (32%). Single-vessel disease accounted for 37% of cases, while the remaining patients exhibited multi-vessel involvement. Nevertheless, only a small proportion of patients had typical angina symptoms (11.8%), and the most frequent complaint was effort dyspnoea (50%). Patients not receiving inhaled corticosteroid therapy were more likely to have extensive coronary artery disease (χ^2^ (6)= 14.228, *p* = 0.027). **Conclusions:** These findings support our hypothesis that atherosclerotic coronary disease is often under-recognised in patients with COPD. ICS-containing therapy appeared to be associated with less extensive coronary artery involvement; however, this observation should be interpreted cautiously.

## 1. Introduction

Chronic obstructive pulmonary disease (COPD) is a common, preventable, and treatable disease, with persistent respiratory symptoms that are aggravated by periodic acute exacerbations. Coronary artery disease (CAD) arises from atherosclerotic lesions within the coronary arteries. COPD and ischaemic heart disease are the most prevalent conditions, ranking among the top three leading causes of death [1]. In patients with chronic obstructive pulmonary disease, coronary artery disease frequently coexists, associated with worse quality of life, increased hospitalisation rate and higher mortality risk [2]. CAD is one of the leading causes of death in patients with COPD [3,4]. Similar risk factors, such as ageing and smoking, contribute to the development of both conditions; however, they do not just have shared risk factors. In recent years, complex interactions have been identified; however, the underlying mechanisms remain unclear. Overlapping symptoms, such as reduced exercise tolerance, dyspnoea and chest discomfort, between ischaemic heart disease and COPD make the differential diagnosis challenging and lead to delayed diagnosis [5]. Current cardiovascular risk stratifications underestimate the ischaemic risk in patients with chronic obstructive pulmonary disease, because risk scoring tools are developed based on data from the general population [6,7]. Although CAD is common at any disease severity, its recognition is often delayed or missed in clinical practice.

Several unresolved questions warrant further investigations. Close cooperation between pulmonologists and cardiologists can lead to more accurate diagnosis and more effective treatment planning. COPD itself is recognised as an independent cardiovascular risk factor and should be included in future cardiovascular risk scoring tools [8,9,10,11]. There is a need to develop new risk stratification tools for the early detection of ischaemic heart disease in this population. Further research is required to develop targeted therapies for improving both conditions.

Given the importance of early detection, we aimed to investigate the prevalence of CAD in patients with COPD. Our research hypothesis was that coronary artery disease is frequently underdiagnosed in this population.

## 2. Materials and Methods

### 2.1. Study Design

We started screening consecutive patients at our heart institute in February 2025. We enrolled patients with moderate and severe COPD in our clinical study. The inclusion criteria were confirmed COPD and the absence of any known cardiac disease in the medical history, except for hypertension. COPD diagnosis was based on the Global Initiative for Chronic Obstructive Lung Disease (GOLD) guidelines and required spirometrically confirmed irreversible airflow obstruction [12]. The exclusion criteria included previous known myocardial infarction, percutaneous coronary intervention (PCI) or coronary artery bypass graft (CABG) surgery, asthma bronchiale, lung transplantation, ongoing acute COPD exacerbation, lung tumour, restrictive lung disease, and age ≤ 18 years. All patients provided written informed consent, and the study was approved by the local ethics committee. During their pulmonary care, patients regularly underwent spirometry tests, completed the COPD Assessment Test (CAT) questionnaire, and chest CT scans were performed in high-risk patients.

In the present study, medical history was obtained, physical examination was performed, and detailed cardiological evaluation was conducted. Blood pressure measurement, 12-lead ECG, and 6 min walk test were performed. Comprehensive laboratory testing was performed, including metabolic, inflammatory, and cardiac biomarkers. In addition to the standard echocardiographic measurements used in daily practice, we performed atrial and ventricular strain analysis using speckle-tracking echocardiography, as well as left and right ventricular 3D measurements with dedicated software. For all examinations, we used the Philips Epiq 7 ultrasound system. 

**Figure 1:**
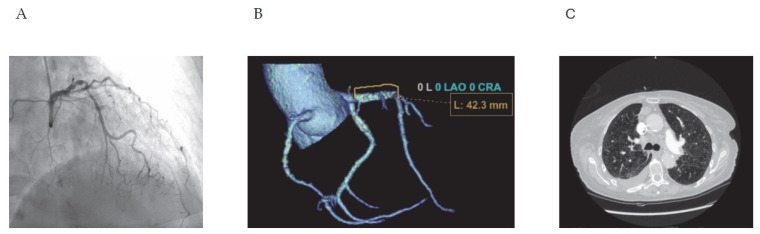
(**A**) Coronary angiography. (**B**) Coronary CT angiography. (**C**) Chest CT.

Three methods were used to detect coronary artery disease: coronary angiography, coronary CT angiography, and calcium score assessment on chest CT (Figure 1).

**Figure 2:**
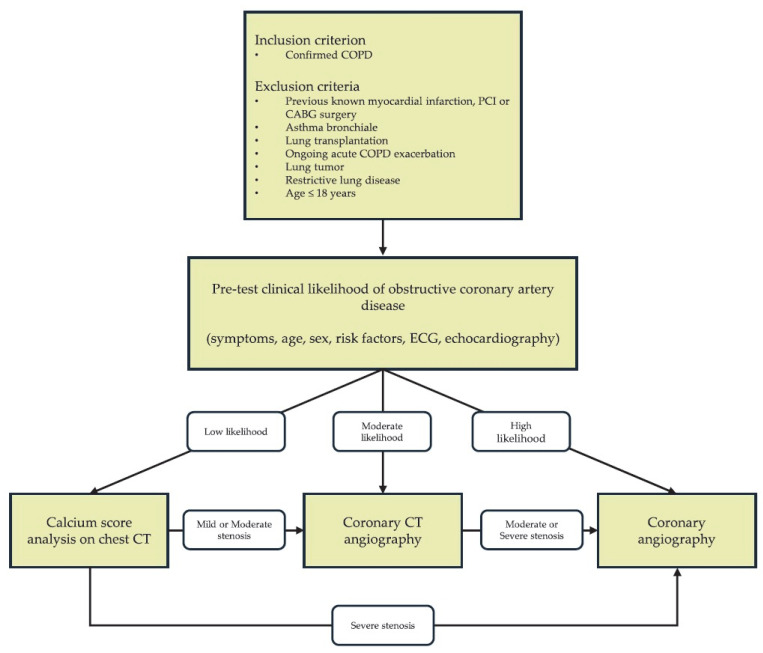
Diagnostic algorithm.

Coronary artery disease was assessed using a stepwise diagnostic algorithm reflecting routine clinical practice (Figure 2).

The pre-test clinical likelihood was determined based on the symptoms, age, sex, cardiovascular risk factors, and comorbidities, in accordance with ESC guidelines for Chronic Coronary Syndromes [13], with electrocardiographic and echocardiographic findings also taken into account during clinical decision making. Patients with very low or low clinical likelihood underwent visual coronary calcium assessment, derived from chest CT performed within the past six months [14,15]. Chest CT calcium findings were recorded using a visual coronary calcium score, categorised as no coronary calcification, mild, moderate, or severe calcification. Patients with moderate likelihood of obstructive CAD underwent ECG-gated coronary CT angiography (CCTA). The presence of atherosclerotic plaques and stenosis severity were evaluated by a radiologist, and categorised as no stenosis, mild (<50%), moderate (50–69%), or severe (≥70%) diameter stenosis. In patients with high or very high clinical likelihood of obstructive CAD, invasive coronary angiography was performed by an interventional cardiologist. All major epicardial vessels, including the left main (LM), left anterior descending (LAD), circumflex artery (Cx), and right coronary artery (RCA), were evaluated. Stenosis severity was graded as no stenosis, mild (<50%), moderate (50–69%), or severe (≥70%), based on the visually estimated percent diameter reduction relative to the reference vessel diameter. When lesion severity was not clearly determinable based on angiography alone, physiological assessment (FFR and/or iFR) was used to support lesion significance and guide revascularisation decisions. Downstream testing followed a pre-specified pathway. Equivocal or moderate-to-severe findings on coronary CT angiography prompted invasive coronary angiography. The presence of mild stenosis on CCTA was accepted as the final diagnostic outcome, and invasive coronary angiography was not performed in accordance with the principle of minimal invasiveness. When chest CT suggested mild or moderate coronary calcification, coronary CT angiography was indicated, whereas patients with severe calcification were referred for invasive coronary angiography. In patients who underwent chest CT, the absence of coronary calcification was classified as ‘no evidence of CAD’ and did not trigger further testing. However, the absence of coronary calcification does not exclude non-calcified plaque; therefore, the prevalence of CAD may be underestimated in the chest CT group.

Coronary artery disease was defined as any evidence of coronary atherosclerosis on any modality, coronary calcification on chest CT, plaque/stenosis on coronary CT angiography, or any angiographic stenosis on coronary angiography. Severity was categorised according to the most advanced finding obtained during diagnostic workup. For harmonised analyses across modalities, we defined anatomically obstructive CAD as stenosis severity of ≥50%, moderate (50–69%) and severe (≥70%). Mild stenosis (<50%) was classified as non-obstructive atherosclerosis. The extent of coronary artery disease was classified as one-, two-, or three-vessel disease, based on the number of major epicardial vessels involved. 

This study follows the STROBE reporting guidelines; the completed STROBE checklist is provided in the Supplementary Materials.

### 2.2. Statistical Analysis

Data were recorded in Microsoft Office Excel, and statistical analyses were performed using SPSS Statistics version 29 (IBM Corp., Armonk, NY, USA).

Continuous variables were reported as mean ± standard deviation. Before examining the differences, the data distribution was evaluated using the Shapiro–Wilk and Kolmogorov–Smirnov tests. For normally distributed variables, an independent samples *t*-test was applied, whereas non-normally distributed data were compared using the Mann–Whitney U test.

Categorical variables were presented as absolute numbers and percentages, and were analysed using the chi-squared test or Fisher’s exact test, as appropriate.

A *p*-value of <0.05 was considered statistically significant in our analyses.

## 3. Results

A total of 76 patients, 39 with moderate COPD (GOLD A and B) and 37 with severe COPD (GOLD E), fulfilling the inclusion criteria were enrolled in our prospective clinical study. The data of the two groups are presented in the first table (Table 1).

There was no significant difference between the two groups in the main risk factors for ischaemic heart disease, such as age, male sex, dyslipidaemia, diabetes mellitus, hypertension, and smoking.

**Figure 3:**
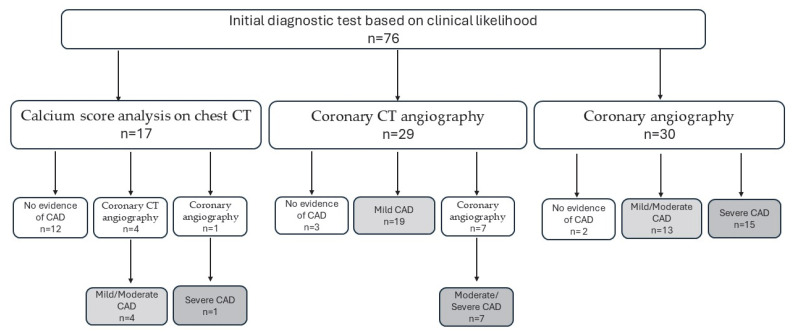
Patient flow through the diagnostic pathway.

In 30 patients, in whom the expected risk of CAD was high, coronary angiography was performed. Coronary CT angiography was conducted in 29 patients and the visual calcium score analysis on chest CT was performed in 17 patients. Based on the diagnostic findings, invasive coronary angiography was performed in seven patients following coronary CT angiography. Chest CT identified severe coronary stenosis in one patient, who subsequently underwent invasive coronary angiography. In addition, coronary CT angiography was indicated in three patients with mild and one patient with moderate calcification detected on chest CT. The number of patients at each diagnostic step is summarised in Figure 3.

Ultimately, coronary angiography was performed in 38 patients, coronary CT in 26 patients, and visual calcium score evaluation on chest CT in 12 patients. Among patients evaluated only by visual coronary calcium assessment on chest CT, no evidence of coronary artery disease was detected. In the subgroup undergoing coronary CT angiography, 11.5% of patients had no detectable coronary artery disease, while 88.5% exhibited mild, non-obstructive coronary stenosis. Among patients referred for invasive coronary angiography, 5.3% had no coronary artery disease, 26.3% had mild stenosis, 18.4% had moderate stenosis, and 50% had severe CAD.

We confirmed the presence of atherosclerotic coronary disease in 59 patients, which represents 78%. Its prevalence was 77% in patients with moderate COPD and 78% in patients with severe COPD. The left main was affected in 8.9%, LAD in 53.6%, Cx in 30.1%, and RCA in 46.4% of the cases. Regarding intravascular lesion distribution, coronary artery disease predominantly involved the proximal segments of all major epicardial vessels. LAD lesions were most frequently located in the proximal segment (63.6%), followed by the mid segment (33.4%), whereas distal involvement was rare (3%). Cx stenoses were confined to the proximal (55.6%) and mid segments (44.4%), with no distal lesions observed. Similarly, in the RCA, the majority of lesions were located in the proximal (59.3%) and mid segments (37.0%), with distal involvement detected in only 3.7% of cases.

Nevertheless, only a small proportion of patients had typical angina symptoms. The most common complaint was effort dyspnoea (50%), atypical chest pain occurred in 26%, 12% were asymptomatic, and typical effort angina pectoris was detected in 12% of cases.

**Figure 4:**
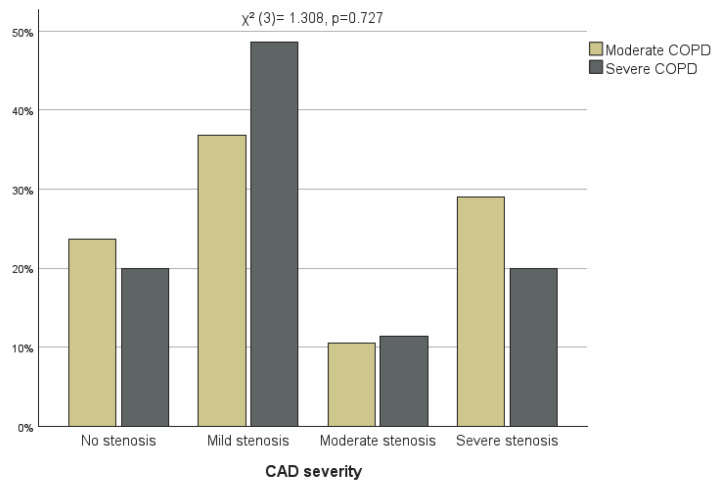
CAD severity in patients with moderate and severe COPD.

**Figure 5:**
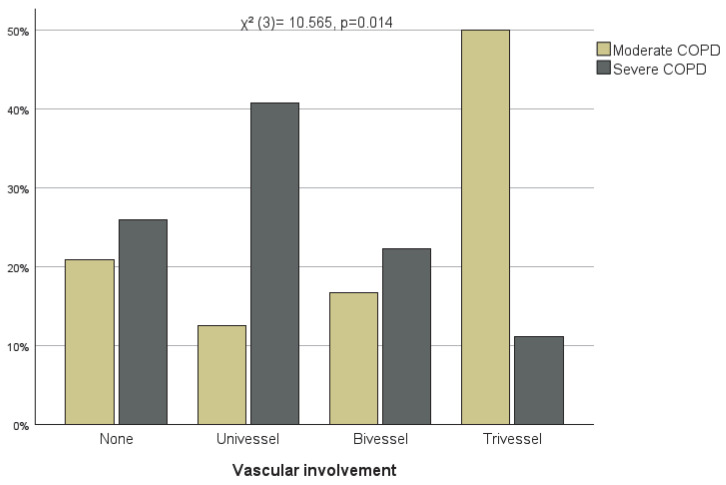
Vascular involvement in patients with moderate and severe COPD.

When comparing patients with moderate and severe COPD, the distribution of CAD severity did not differ significantly (χ^2^ (3)= 1.308, *p* = 0.727). Minor visual differences can be observed across categories (Figure 4). In contrast, the extent of coronary artery disease differed significantly between the two groups (χ^2^ (3)= 10.565, *p* = 0.014), being more severe in patients with moderate COPD (Figure 5).

**Figure 6:**
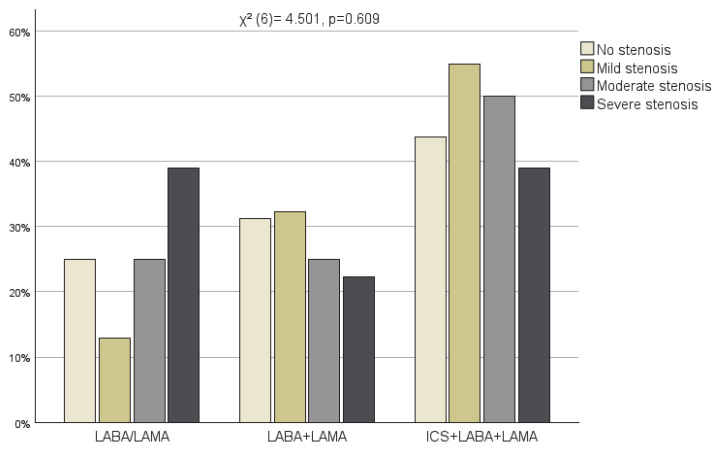
COPD therapy and CAD severity.

**Figure 7:**
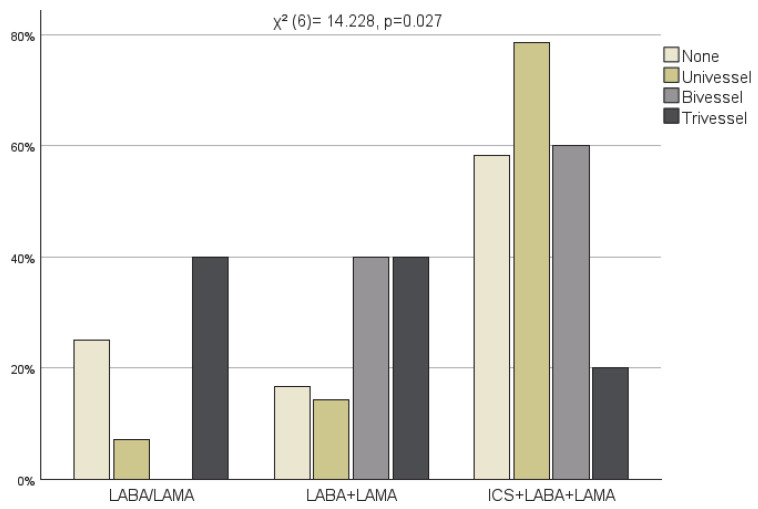
COPD therapy and vascular involvement.

COPD therapy − including LABA (Long-acting beta2-agonist)/LAMA (Long-acting muscarinic antagonist), LABA + LAMA, and ICS (Inhaled corticosteroid) + LABA + LAMA−, and CAD severity were also compared. There was no statistically significant association between the degree of coronary artery stenosis and the use of these medication classes (χ^2^ (6)= 4.501, *p* = 0.609) (Figure 6). However, a significant difference was observed in the extent of coronary artery disease across the COPD medication groups (χ^2^ (6)= 14.228, *p* = 0.027) (Figure 7). While in the LABA/LAMA and LABA + LAMA groups, the prevalence of trivessel disease was the highest, the ICS + LABA + LAMA group showed a marked shift toward univessel and bivessel involvement.

## 4. Discussion

Over 80% of patients with COPD have at least one comorbid chronic disease, of which cardiovascular diseases have one of the highest prevalences [16]. The most common causes of death in patients with COPD are respiratory or cardiovascular diseases. Approximately 39% of deaths are attributable to cardiovascular disease [17]. Atherosclerotic coronary disease occurrence was high in our COPD population, and we confirmed the disease in approximately four out of five patients (78%). In contrast, previous studies have reported a prevalence of 7% to 33% in this population [18,19,20], but high-level evidence is still lacking. Differences in the reported CAD prevalence across studies are partly explained by heterogeneity in case definitions and diagnostic strategies. In the present study, coronary artery disease was defined as any detectable coronary atherosclerosis, whereas prior reports often focused on clinically manifest or obstructive disease. Moreover, in many earlier studies, CAD identification relied on cardiovascular risk-driven testing or administrative data. Contemporary cardiovascular risk stratification tools are known to underestimate cardiovascular risk in patients with COPD, which may lead to underdiagnosis in routine clinical practice. In contrast, all patients in our study underwent coronary imaging as part of a structured diagnostic workup.

Acute coronary syndrome (ACS) can be the first symptom of coronary artery disease in patients with COPD. COPD exacerbations have been shown to increase the risk of cardiovascular events. This increased risk occurs particularly within the first 30 days, but it remains significantly higher for up to one year post-exacerbation [21]. These findings raise the question of whether early cardiovascular screening should be considered in patients with COPD. Overlapping symptoms between CAD and COPD make the differential diagnosis challenging and lead to delayed diagnosis [5]. Symptoms such as dyspnoea, chest tightness, fatigue, and reduced exercise tolerance are common to both diseases. Many patients with established COPD may attribute the worsening of their respiratory symptoms solely to their pulmonary disease. Only 12% of our patients had typical angina pectoris; the most common complaint was effort dyspnoea. This may be explained by neuropathy observed in patients with COPD, which could account for undiagnosed coronary artery disease. The exact mechanisms are still being researched, and it is thought that chronic hypoxia, systemic inflammation, and smoking contribute to nerve damage [22,23,24]. Similar to patients treated for diabetes mellitus, coronary artery disease may also be present in the absence of typical angina symptoms, making the diagnosis even more challenging.

### 4.1. Ischaemia with Non-Obstructive Coronary Arteries (INOCA)

Our study focused on coronary artery stenosis affecting the epicardial vessels. However, the contemporary cardiovascular literature highlights the importance of ischaemia with non-obstructive coronary arteries (INOCA). In this condition, the mismatch between perfusion and myocardial oxygen consumption can lead to myocardial ischaemia [13].

Coronary microvascular dysfunction (CMD) represents one of the principal pathophysiological mechanisms underlying ischaemia with non-obstructive coronary arteries [25]. Chronic inflammatory conditions, such as COPD, have been shown to be associated with CMD [26]. These patients may exhibit wide variation in clinical presentation and symptom burden. Symptoms are often solely attributed to pulmonary limitation. Standard anatomical imaging modalities used in routine clinical practice have limited ability to detect CMD. The diagnosis of coronary microvascular dysfunction (CMD) requires a structured approach. The absence of obstructive epicardial coronary artery disease and objective evidence of myocardial ischaemia should be demonstrated. Functional imaging modalities, including stress echocardiography, PET, perfusion CCTA, and cardiac magnetic resonance (CMR), may facilitate diagnosis by measuring the coronary flow reserve (CFR) [27]. During invasive coronary angiography, measurements of CFR and microvascular resistance may allow for an accurate diagnosis [13].

In our cohort, only 12% of patients were asymptomatic, and a substantial proportion of the patients were found to have non-obstructive coronary artery disease. This dissociation between symptom burden and angiographic stenosis severity suggests that mechanisms beyond focal epicardial obstruction may contribute to myocardial ischaemia and symptom generation in patients with COPD. Although our study was not designed to assess microvascular function and did not include dedicated functional testing, the high prevalence of non-obstructive coronary disease and the predominance of dyspnoea-driven symptoms underscore the potential clinical relevance of CMD in COPD. Future studies incorporating functional coronary assessment are warranted.

Taken together, consideration of CMD and INOCA provide a broader and more contemporary framework for interpreting the high prevalence of coronary atherosclerosis and the often -typical clinical presentation observed in patients with COPD. Recognising these mechanisms may have important implications for diagnostic strategies and therapeutic decision making beyond the traditional focus on epicardial stenosis.

### 4.2. Treatment, Follow Up

In patients with coronary artery disease, lifestyle modification and cardiovascular risk factor management were initiated or reinforced. In addition, guideline-directed optimal medical therapy was implemented, including antianginal treatment and disease-modifying pharmacotherapy, such as statins and antiplatelet agents. Medication changes were not systematically recorded in this dataset.

Coronary revascularisation was performed in nine patients, including one patient who underwent coronary artery bypass grafting and eight patients who were treated with percutaneous coronary intervention. Among patients undergoing revascularisation, RCA involvement was the most common, followed by LAD and Cx involvement. Only one patient presented with significant LM stenosis. Cardiac magnetic resonance imaging was performed prior to revascularisation in three patients to assess myocardial viability and scar burden. CMR provides complementary functional information beyond anatomical coronary imaging and helps distinguish ischaemic scar from alternative causes of symptoms [13,28].

Although follow-up outcome data are not yet available, longitudinal follow-up of this cohort has been planned. Future analyses will focus on changes in quality of life, symptom burden, and hard clinical endpoints, including all-cause and cardiovascular mortality.

### 4.3. Inhaled Corticosteroid Therapy

Treatments for one condition may influence the other; these patients require holistic care. Most treatment strategies continue to focus on individual disease silos, rather than acknowledging the multifaceted interplay between pulmonary and cardiovascular health. The pathophysiological mechanisms of these two diseases are multifactorial and interconnected. The potential mechanisms behind their coexistence remain unclear, but several pathways play roles in the development of coronary artery disease in patients with COPD. The three main potential underlying pathomechanisms are systemic inflammation, lung hyperinflation, and sustained or intermittent hypoxia [29,30,31]. Improving lung function and reducing exacerbations can lead to decreases in inflammation, hyperinflation and hypoxia. Therefore, optimal COPD therapy may improve cardiac status.

Contrary to expectations, patients with moderate COPD had more extensive coronary artery disease than those with severe COPD. In our study population, patients with severe COPD (GOLD E) received triple therapy, a combination of ICS + LABA + LAMA according to GOLD recommendations [12]. Patients not receiving inhaled corticosteroid therapy were observed to have more extensive coronary artery disease. Numerous clinical trials have demonstrated the beneficial effects of ICS. The SUMMIT trial (Survival in Chronic Obstructive Pulmonary Disease with Heightened Cardiovascular Risk) investigated the effect of ICS and LABA combination in patients with moderate COPD and found that this therapy reduced the rate of COPD exacerbations [32]. The IMPACT study compared triple therapy (ICS + LABA + LAMA) with two dual therapies (ICS + LABA or LABA + LAMA). Triple therapy reduced the incidences of exacerbations, hospitalisations, and all-cause mortality [33]. The ETHOS trial (Efficacy and Safety of Triple Therapy in Obstructive Lung Disease) compared two doses of triple therapy versus two dual therapies (LAMA + LABA and LABA + ICS). Triple therapy with high-dose ICS was associated with reduced cardiovascular mortality and exacerbation rates [34]. The ETHOS study demonstrated the cardiovascular benefits of higher-dose ICS therapy by reducing cardiovascular mortality. In our study, we observed an association between ICS use and lower extent of stable coronary artery disease. The likely mechanism is the attenuation of systemic inflammation. ICS reduces airway inflammation and can reduce systemic inflammatory activity, too [35,36,37]. Both COPD and CAD share a pathophysiological mechanism in which low-grade systemic inflammation plays an important role. However, this observation must be interpreted with caution, as ICS use in our study was closely linked to COPD severity and, therefore, subject to substantial confounding. Therefore, the association between ICS use and the extent of CAD should be considered strictly hypothesis-generating. The potential interaction between ICS therapy and coronary artery disease warrants further investigation in prospective randomised clinical trials.

### 4.4. Clinical Relevance

Coronary artery disease is often undiagnosed in patients with COPD. Therefore, active screening for CAD should be considered in this population. ICS therapy appears to improve pulmonary outcomes and reduce mortality in COPD; however, its potential cardiovascular effects, including a possible association with less extensive coronary artery disease, remain speculative and require confirmation in dedicated randomised studies.

### 4.5. Limitations

Some limitations of this study should be considered when interpreting the results. The sample size was small and the study population was limited to a single healthcare centre, which may limit the generalisability of the findings to other settings. Due to limitations in sample size, multivariable or propensity score-based adjustment was not feasible. Although all patients underwent structured coronary assessment using invasive or non-invasive modalities according to current guidelines, the choice of diagnostic test was risk-based. Therefore, a degree of work-up and verification bias cannot be excluded. Furthermore, in patients classified as having no evidence of coronary artery disease based on visual calcium assessment on chest CT, the presence of non-calcified atherosclerotic plaque cannot be fully excluded, potentially leading to an underestimation of CAD. Nevertheless, we are convinced that the results of the analysis obtained in this study can serve as a reference for future research in this field. Prospective, multicentre studies with comprehensive clinical data collection would be valuable for confirming the current findings.

## 5. Conclusions

The global prevalences of COPD and CAD appear to be substantial. The intersection of chronic obstructive pulmonary disease and ischaemic heart disease presents a notable clinical challenge because of their complex pathophysiology and overlapping symptoms. Traditional symptom-based diagnostic approaches may be less accurate in identifying CAD in patients with COPD. Current guidelines are mostly restricted to the management of individual diseases. However, our findings should be interpreted cautiously and primarily serve to generate hypotheses for future prospective and randomised studies, due to the limitations of our study. Future diagnostic and therapeutic strategies integrating pulmonary and cardiovascular assessment may lead to improved quality of life and reduced mortality in this population.

## Figures and Tables

**Table 1:** Clinical characteristics of the study population.

	All Patients (n = 76)	Moderate COPD (n = 39)	Severe COPD (n = 37)	*p* Value
Age (year)	66 ± 8	64 ± 10	67 ± 6	0.121
Male	34 (45%)	19 (49%)	15 (41%)	0.540
Body mass index (kg/m^2^)	27.1 ± 5.7	28.0 ± 5.7	26.2 ± 5.4	0.183
Smoking (pack/year)	34.5 ± 24.4	38.0 ± 26.4	30.9 ± 21.4	0.212
Hypertension	52 (68%)	26 (67%)	26 (70%)	>0.999
Diabetes mellitus	16 (21%)	9 (23%)	7 (19%)	0.740
CAT score	18.0 ± 7.4	15.2 ± 6.5	21.0 ± 7.2	<0.001
6 min walk test (metre)	368.5 ± 96.1	405.6 ± 61.9	329.4 ± 109.1	<0.001
Spirometry test				
Forced Expiratory Volume in 1 s (%)	54.5 ± 17.3	63.4 ± 14.6	45.2 ± 14.6	<0.001
Forced Vital Capacity (%)	79.1 ± 18.8	85.3 ± 16.2	72.6 ± 19.2	0.002
Forced Expiratory Volume in 1 s/Forced Vital Capacity (%)	55.8 ± 9.7	59.9 ± 9.0	51.3 ± 8.5	<0.001
COPD therapy				
LABA	3 (4%)	3 (8%)	0 (0%)	0.240
LAMA	14 (18%)	14 (36%)	0 (0%)	<0.001
LABA + LAMA	22 (29%)	22 (56%)	0 (0%)	<0.001
ICS + LABA + LAMA	37 (49%)	0 (0%)	37 (100%)	<0.001
Cardiovascular medication				
Renin-angiotensin-aldosterone system inhibitor	44 (58%)	23 (59%)	21 (57%)	0.990
Calcium channel blocker	36 (47%)	19 (49%)	17 (46%)	0.823
Thiazide diuretics	22 (29%)	9 (23%)	13 (35%)	0.314
β blocker	36 (47%)	20 (51%)	16 (43%)	0.501
ECG				
Repolarisation abnormalities	22 (29%)	12 (31%)	10 (27%)	0.740
Conduction disturbances	9 (12%)	5 (13%)	4 (11%)	0.880
Extrasystole	8 (11%)	4 (10%)	4 (11%)	>0.999
Laboratory parameters				
Cholesterol (mmol/L)	5.0 ± 1.2	4.9 ± 1.2	5.2 ± 1.2	0.232
Low-Density Lipoprotein (mmol/L)	2.9 ± 1.0	2.8 ± 1.1	3.1 ± 1.0	0.174
Triglyceride (mmol/L)	2.0 ± 1.4	2.3 ± 1.7	1.8 ± 1.1	0.141
Hemoglobin A1c (%)	6.2 ± 0.7	6.3 ± 0.9	6.1 ± 0.5	0.200
Estimated Glomerular Filtration Rate (ml/min/1.7 m^2^)	76.0 ± 15.5	77.0 ± 14.9	74.9 ± 16.5	0.571
Creatine kinase (U/L)	142.6 ± 172.1	163.4 ± 222.5	120.7 ± 87.9	0.280
Troponin (ng/L)	10.0 ± 7.5	9.0 ± 6.8	11.1 ± 8.0	0.223
Lactate (mmol/L)	1.7 ± 0.8	1.7 ± 0.8	1.7 ± 0.7	0.958
Transferrin saturation (%)	22.1 ± 12.5	22.7 ± 14.4	21.4 ± 10.0	0.661
Ferritin (ug/L)	162.4 ± 142.6	143.2 ± 142.9	182.7 ± 139.4	0.237
C-reactive protein (mg/L)	4.7 ± 6.7	3.9 ± 3.5	5.6 ± 8.8	0.269
D-dimer (ug/L)	999.7 ± 2184.8	1267.9 ± 2940.1	700.0 ± 509.3	0.270
Eosinophil count (G/L)	0.2 ± 0.1	0.2 ± 0.2	0.2 ± 0.1	0.128
Interleukin 6 (pg/mL)	6.5 ± 7.4	6.5 ± 5.4	6.4 ± 9.1	0.970
Nt-proBNP (pg/mL)	222.0 ± 254.6	186.5 ± 161.8	259.4 ± 321.9	0.213
Echocardiography				
Right ventricular systolic pressure (mmHg)	40.3 ± 12.1	37.8 ± 14.8	42.5 ± 9.0	0.343
Right ventricular wall thickness (mm)	6.5 ± 1.5	6.8 ± 1.6	6.2 ± 1.3	0.068
Tricuspid Annular Plane Systolic Excursion (mm)	22.3 ± 3.5	22.6 ± 3.3	22.0 ± 3.8	0.451
Right ventricular ejection fraction (%)	46.0 ± 7.1	46.5 ± 7.8	45.4 ± 6.3	0.559
Right ventricular end-diastolic volume (mL)	97.9 ± 33.5	100.7 ± 32.3	94.7 ± 35.2	0.496
Right ventricular end-systolic volume (mL)	52.8 ± 19	54.1 ± 19.7	51.1 ± 18.4	0.550
Right ventricular stroke volume (mL)	45.2 ± 17.2	46.4 ± 15.4	43.5 ± 19.3	0.508
Right ventricular free wall strain (%)	−23.5 ± 6.7	−23.3 ± 7.0	−23.7 ± 6.5	0.793
Left ventricular ejection fraction (%)	59.0 ± 5.1	58.0 ± 5.9	60.1 ± 3.7	0.063
Preserved ejection fraction (EF > 50%)	70 (92%)	34 (87%)	36 (97%)	0.160
Mildly reduced ejection fraction (EF 41–49%)	6 (8%)	5 (13%)	1 (3%)	0.200
Reduced ejection fraction (EF < 40%)	0 (0%)	0 (0%)	0 (0%)	N/A
Left ventricular end-diastolic volume (mL)	107.7 ± 33.8	111.8 ± 36.2	103.2 ± 31.0	0.322
Left ventricular end-systolic volume (mL)	47.0 ± 18.3	49.6 ± 18.8	44.1 ± 17.6	0.238
Left ventricular stroke volume (mL)	60.7 ± 17.9	62.1 ± 19.8	59.1 ± 15.6	0.504
Left ventricular global longitudinal strain (%)	−16.1 ± 4.8	−16.4 ± 3.7	−15.8 ± 5.7	0.600
Left ventricular global circumferential strain (%)	−26.4 ± 4.8	−26.1 ± 5.0	−26.8 ± 4.5	0.517
Left atrial area (cm^2^)	15.5 ± 4.3	16.3 ± 3.8	14.6 ± 4.7	0.100
Left atrial reservoir strain (%)	34.7 ± 12.9	36.0 ± 14.4	33.4 ± 11.2	0.386
Right atrial area (cm^2^)	14.8 ± 4.2	15.4 ± 4.3	14.3 ± 4.1	0.265
Right atrial reservoir strain (%)	32.7 ± 14.4	34.8 ± 13.6	30.6 ± 14.8	0.205
Coronary artery disease				
CAD prevalence	59 (78%)	30 (77%)	29 (78%)	0.853
Symptoms				
Effort dyspnoea	30 (50%)	16 (53%)	14 (48%)	0.710
Effort angina pectoris	7 (12%)	2 (7%)	5 (17%)	0.390
Atypical chest pain	15 (26%)	5 (17%)	10 (35%)	0.210
Asymptomatic	7 (12%)	7 (23%)	0 (0%)	0.009
Lesion severity				0.727
Non-obstructive (<50%)	33 (56%)	15 (50%)	18 (62%)	
Moderate (50–69%)	7 (12%)	3 (10%)	4 (14%)	
Severe (≥70%)	19 (32%)	12 (40%)	7 (24%)	
Agatston score	288.0 ± 502.3	327.3 ± 340.6	278.0 ± 402.3	0.651
Vascular involvement				0.014
Univessel disease	22 (37%)	5 (17%)	17 (59%)	
Bivessel disease	14 (24%)	6 (20%)	8 (27%)	
Trivessel disease	23 (39%)	19 (63%)	4 (14%)	

## Data Availability

The data presented in this study are available on request from the corresponding author. The data are not publicly available due to privacy and ethical restrictions.

## Supplementary Materials

The following supporting information can be downloaded at: https://www.mdpi.com/article/10.3390/jcm15051896/s1, Table S1: STROBE checklist of items that should be included in reports of cross-sectional studies.

## Abbreviations

The following abbreviations are used in this manuscript:

ACS	Acute Coronary Syndrome
CABG	Coronary Artery Bypass Grafting
CAD	Coronary artery disease
CAT	COPD Assessment Test
CCTA	Coronary CT Angiography
CFR	Coronary Flow Reserve
CMD	Coronary Microvascular Dysfunction
CMR	Cardiac Magnetic Resonance
COPD	Chronic Obstructive Pulmonary Disease
CT	Computed Tomography
Cx	Circumflex artery
ECG	Electrocardiography
ESC	European Society of Cardiology
GOLD	Global Initiative for Chronic Obstructive Lung Disease
ICS	Inhaled corticosteroid
INOCA	Ischaemia with Non-Obstructive Coronary arteries
LABA	Long-acting Beta 2-agonist
LAD	Left anterior descending
LAMA	Long-acting muscarinic antagonist
LM	Left main
PCI	Percutaneous Coronary Intervention
RCA	Right coronary artery
