# Supplementary material for: Undiagnosed Coronary Artery Disease in Patients with COPD
Source: J Clin Med. 2026 Mar 2;15(5):1896. doi: 10.3390/jcm15051896 (PMC12986395; doi:10.3390/jcm15051896)
Supplement: Supplementary file 1 [file jcm-15-01896-s001.zip › jcm-4157731-supplementary.pdf]

Table S1: STROBE Statement—Checklist of items that should be included in reports of ***cross-sectional studies***

	Item No	Recommendation	
**Title and abstract**	1	(*a*) Indicate the study's design with a commonly used term in the title or the abstract	Abstract
		(*b*) Provide in the abstract an informative and balanced summary of what was done and what was found	Abstract
**Introduction**			
Background/rationale	2	Explain the scientific background and rationale for the investigation being reported	Introduction
Objectives	3	State specific objectives, including any prespecified hypotheses	Introduction
**Methods**			
Study design	4	Present key elements of study design early in the paper	Patients and Methods, Study design
Setting	5	Describe the setting, locations, and relevant dates, including periods of recruitment, exposure, follow-up, and data collection	Patients and Methods, Study design
Participants	6	(*a*) Give the eligibility criteria, and the sources and methods of selection of participants	Patients and Methods, Study design
Variables	7	Clearly define all outcomes, exposures, predictors, potential confounders, and effect modifiers. Give diagnostic criteria, if applicable	Patients and Methods, Study design
Data sources/ measurement	8*	For each variable of interest, give sources of data and details of methods of assessment (measurement). Describe comparability of assessment methods if there is more than one group	Patients and Methods, Study design
Bias	9	Describe any efforts to address potential sources of bias	Patients and Methods, Study design, Limitations
Study size	10	Explain how the study size was arrived at	Results, Study population
Quantitative variables	11	Explain how quantitative variables were handled in the analyses. If applicable, describe which groupings were chosen and why	Patients and Methods, Statistical analysis
Statistical methods	12	(*a*) Describe all statistical methods, including those used to control for confounding	Patients and Methods, Statistical analysis
		(*b*) Describe any methods used to examine subgroups and interactions	Patients and Methods, Statistical analysis
		(*c*) Explain how missing data were addressed	Patients and Methods, Statistical analysis
		(*d*) If applicable, describe analytical methods taking account of sampling strategy	Patients and Methods, Statistical analysis
		(*e*) Describe any sensitivity analyses	Patients and Methods, Statistical analysis
**Results**			

Participants	13*	(a) Report numbers of individuals at each stage of study—eg numbers potentially eligible, examined for eligibility, confirmed eligible, included in the study, completing follow-up, and analysed	Results, Study population
		(b) Give reasons for non-participation at each stage	Patients and Methods, Study design
		(c) Consider use of a flow diagram	N/A
Descriptive data	14*	(a) Give characteristics of study participants (eg demographic, clinical, social) and information on exposures and potential confounders	Results, Study population
		(b) Indicate number of participants with missing data for each variable of interest	N/A
Outcome data	15*	Report numbers of outcome events or summary measures	Results
Main results	16	(*a*) Give unadjusted estimates and, if applicable, confounder-adjusted estimates and their precision (e.g., 95% confidence interval). Make clear which confounders were adjusted for and why they were included	N/A
		(*b*) Report category boundaries when continuous variables were categorised	Results, Study population
		(*c*) If relevant, consider translating estimates of relative risk into absolute risk for a meaningful time period	N/A
Other analyses	17	Report other analyses done—eg analyses of subgroups and interactions, and sensitivity analyses	Results
**Discussion**			
Key results	18	Summarise key results with reference to study objectives	Discussion, Clinical relevance
Limitations	19	Discuss limitations of the study, taking into account sources of potential bias or imprecision. Discuss both direction and magnitude of any potential bias	Limitations
Interpretation	20	Give a cautious overall interpretation of results considering objectives, limitations, multiplicity of analyses, results from similar studies, and other relevant evidence	Discussion
Generalisability	21	Discuss the generalisability (external validity) of the study results	Conclusion
**Other information**			
Funding	22	Give the source of funding and the role of the funders for the present study and, if applicable, for the original study on which the present article is based	No funding was received for this study.

*Give information separately for exposed and unexposed groups.

Note: An Explanation and Elaboration article discusses each checklist item and gives methodological background and published examples of transparent reporting. The STROBE checklist is best used in conjunction with this article (freely available on the Web sites of PLoS Medicine at http://www.plosmedicine.org/, Annals of Internal Medicine at

http://www.annals.org/, and Epidemiology at http://www.epidem.com/). Information on the STROBE Initiative is available at www.strobe-statement.org.
